# Artificial intelligence-augmented analysis of contemporary procedural, mortality, and cost trends in carcinoid heart disease in a large national cohort with a focus on the “forgotten pulmonic valve”

**DOI:** 10.3389/fcvm.2022.1071138

**Published:** 2023-02-08

**Authors:** Dominique J. Monlezun, Andrew Badalamenti, Awad Javaid, Kostas Marmagkiolis, Kevin Honan, Jin Wan Kim, Rishi Patel, Bindu Akhanti, Dan Halperin, Arvind Dasari, Efstratios Koutroumpakis, Peter Kim, Juan Lopez-Mattei, Syed Wamique Yusuf, Mehmet Cilingiroglu, Mamas A. Mamas, Igor Gregoric, James Yao, Saamir Hassan, Cezar Iliescu

**Affiliations:** ^1^Department of Cardiology, The University of Texas M.D. Anderson Cancer Center, Houston, TX, United States; ^2^Center for Artificial Intelligence and Health Equities, Global System Analytics and Structures (GSAS), New Orleans, LA, United States; ^3^Division of Cardiovascular Medicine, The University of Texas Health Sciences Center at Houston, Houston, TX, United States; ^4^Division of Cardiovascular Medicine, Kirk Kerkorian School of Medicine at the University of Nevada-Las Vegas, Las Vegas, NV, United States; ^5^Division of Cardiovascular Disease, University of Arkansas for Medical Sciences, Little Rock, AR, United States; ^6^Keele Cardiovascular Research Group, Keele University, Stoke-on-Trent, United Kingdom

**Keywords:** cardio-oncology, carcinoid, valvular disease, artificial intelligence, propensity score

## Abstract

**Background:**

Carcinoid heart disease is increasingly recognized and challenging to manage due to limited outcomes data. This is the largest known cohort study of valvular pathology, treatment (including pulmonary and tricuspid valve replacements [PVR and TVR]), dispairties, mortality, and cost in patients with malignant carcinoid tumor (MCT).

**Methods:**

Machine learning-augmented propensity score-adjusted multivariable regression was conducted for clincal outcomes in the 2016–2018 U.S. National Inpatient Sample (NIS). Regression models were weighted by the complex survey design and adjusted for known confounders and the likelihood of undergoing valvular procedures.

**Results:**

Among 101,521,656 hospitalizations, 55,910 (0.06%) had MCT. Patients with MCT vs. those without had significantly higher inpatient mortality (2.93 vs. 2.04%, *p* = 0.002), longer mean length of stay (12.20 vs. 4.62, *p* < 0.001), and increased mean total cost of stay ($70,252.18 vs. 51,092.01, *p* < 0.001). There was a ste*p-*wise increased rate of TVR and PVR with each subsequent year, with significantly more TV (0.16% vs. 0.01, *p* < 0.001) and PV (0.03 vs. 0.00, *p* = 0.040) diagnosed with vs. without MCT for 2016, with comparable trends in 2017 and 2018. There were no significant procedural disparities among patients with MCT for sex, race, income, urban density, or geographic region, except in 2017, when the highest prevalence of PV procedures were performed in the Western North at 50.00% (*p* = 0.034). In machine learning and propensity score augmented multivariable regression, MCT did not significantly increase the likelihood of TVR or PVR. In sub-group analysis restricted to MCT, neither TVR nor PVR significantly increased mortality, though it did increase cost (respectively, $141,082.30, *p* = 0.015; $355,356.40, *p* = 0.012).

**Conclusion:**

This analysis reflects a favorable trend in recognizing the need for TVR and PVR in patients with MCT, with associated increased cost but not mortality. Our study also suggests that pulmonic valve pathology is increasingly recognized in MCT as reflected by the upward trend in PVRs. Further research and updated societal guidelines may need to focus on the “forgotten pulmonic valve” to improve outcomes and disparities in this understudied patient population.

## 1. Introduction

Carcinoid tumors are rare neuroendocrine tumors (NETs) that arise most commonly in the lung and gastrointestinal tract, with an incidence of 5–7 per 100,000 people (1, 2). Metastatic NETs can result in carcinoid syndrome due to the ectopic production of vasoactive hormones, including serotonin, histamine, bradykinin, tachykinins, and prostaglandins that lead to the classic features of carcinoid syndrome, including flushing, bronchospasm, and diarrhea (3).

Carcinoid heart disease (CHD) occurs in up to 50% of patients with carcinoid syndrome and is the initial presentation in up to 20% of patients (4), most commonly involving the tricuspid and pulmonic valves. Severe tricuspid regurgitation (TR) and pulmonic regurgitation (PR), less commonly stenosis, lead to symptoms of right-sided heart failure and increased mortality. The rarity of the disease and the limited dedicated societal guidelines makes management of these complex patients challenging. The objective of this study was to evaluate the national trends in MCT in relation to valvular pathology, repair and replacements (with additional focus on pulmonic valve), disparities, and outcomes using a large national database.

## 2. Methods

### 2.1. Study design

This is the first known artificial intelligence and propensity score-supported nationally representative longitudinal multicenter analysis of inpatient mortality and total cost among hospitalized adults based on malignant carcinoid tumor (MCT) in relation to valvular pathology, inpatient valvular procedure (repair and replacement), disparities, mortality, and cost outcomes. The 2016, 2017, and 2018 NIS datasets were selected for this study as they are among the latest available datasets and the first to use ICD-10 coding and so better reflect current clinical trends in diagnoses and procedures compared to prior years. Study inclusion criteria included all NIS hospitalizations for adults age 18 years or older during the above index time periods. This study used de-identified data and was conducted according to the ethical principles in the Declaration of Helsinki and the regulatory standards of the nation of origin (and thus did not require Institutional Review Board review, exempt determination, or informed consent as confirmed by the United States National Bureau of Economic Research and the Department of Health and Human Services' Agency for Healthcare Research and Quality [AHRQ]).

### 2.2. Data source

The data source for this study was the largest all-payer inpatient administrative dataset in the United States, the National Inpatient Sample (NIS), sponsored by the AHRQ and maintained within the Healthcare Cost and Utilization Project (HCUP). The NIS includes ~1 of every 5 hospital discharges. To reduce sampling bias, the sampling strategy has been modified in the most recent data to produce results more generalizable to all inpatient discharges in the country. The dataset includes demographic, comorbidity, procedural, mortality, length of stay, and cost for each adult hospitalization.

### 2.3. Bivariable statistical analysis

Descriptive statistics and bivariable analysis by MCT was performed for the full sample and in sub-group analysis by year (2016, 2017, and 2018). Comorbidities were selected for analysis (and identified in the dataset by their ICD-10 codes) based on their clinical and/or statistical significance identified in prior published studies and current clinical practice. The comorbidities included in this study were hypertension (HTN), diabetes (DM), coronary artery disease (CAD), smoking, congestive heart failure (CHF), chronic kidney disease (CKD), and valvular disease. For continuous variables, independent sample *t*-tests were performed to compare means and Wilcoxon rank sum tests were performed for medians. For categorical variables, Pearson chi square tests or Fisher exact tests were performed to compare proportions as applicable.

### 2.4. Regression statistical analysis, machine learning analysis, and model optimization

The primary outcome was mortality and the secondary outcomes were valvular procedure (repair/replacement for tricuspid valve [TV], pulmonary valve [PV], mitral valve [MV], and aortic valve [AV]), length of stay (in days), and total cost (in U.S. dollars [$]).

To maximize the likelihood of valid (externally and internally) and replicable results, regression model performance was optimized according to the following sequential process. First, variables were that were clinically or statistically significant were identified in the existing literature, clinic practice, and bivariable analysis to be considered in the final regression models. Second, those variables were included in forward and backward stepwise regression to augment decision-making on the variables ultimately included in the final regression models. Third, the regression results were compared to those generated by backward propagation neural network machine learning to ensure comparability by root mean squared error and accuracy. Fourth, regression model performance was additionally assessed with correlation matrix, area under the curve, Hosmer-Lemeshow goodness-of-fit test, Akaike and Schwarz Bayesian information criterion, variance inflation factor, and tolerance, multicollinearity, and specification error. Fifth, the models were re-run continually with fine tuning the final models and final variables until the above process confirmed optimal performance was reached. Based on the above process, all regression models were ultimately adjusted for age, race, income, metastases, CHF, HTN, and mortality risk as calculated by the NIS according to diagnosis-related group (DRG). Other variables were excluded based upon the machine learning analysis and diagnostic testing to produce the most clinically and statistically justifiable models.

### 2.5. Bayesian machine learning-augmented propensity score translational (BAM-PS) statistics

Sub-group analysis within MCT was then conducted using the above process within BAM-PS. This novel hybrid analytic methodology leverages the synergistic advantages of three methodological components by integrating them with each other: (a) ML-PSr (Machine Learning-augmented Propensity Score adjusted multivariable regression), in which the traditional statistical methodology of causal inference-based propensity score analysis is augmented (b) by ML capable of handling higher dimensional, more complex, and faster data streams, and then translates its results as informative priors for (c) Bayesian regression (5–14). BAM-PS seeks to preserve internal validity in analytic methodology while expanding it (i.e. by reducing the likelihood of relevant omitted variables) and its external validity (by increasing generalizability through greater number of data sources to more accurately and precisely reflect real-world clinical practice in real-time for more timely, accurate, precise, and relevant predictions to augment organizational and clinical decision making in the AI-augmented and transforming healthcare systems). BAM-PS enables both direct (through integration) and indirect (through informative priors) linked datasets and data streams, including combining smaller, more granular datasets with larger, more generalizable datasets. This methodology thus is meant to further provide a more effective and efficient analytics means that better approximates the real-time and more complex distributed cloud-based data collection, computing, and informed decision-making in the emerging model of healthcare as an integrated digital health ecosystem; this ecosystem leverages and integrates diverse partners (healthcare systems, public health systems, technology companies, governments, and community organizations, etc.) to optimize equitable value-based healthcare and societal wellbeing, particularly through AI-enabled Big Data and the Internet of Things, all within the global digital ecosystem generated by the Fourth Industrial Revolution (9). Traditional statistics alone are therefore insufficient for the scope, speed, and complexity of the reality of modern healthcare increasingly, while AI alone lacks the broad understanding and acceptance of the medical community. BAM-PS accordingly utilizes both domains of analytics together within the larger integrated AI-driven Computational Ethics and policy analysis (AiCE), previously demonstrated for real-time clinical decision support through integration with electronic health records and clinical and organizationl work-flows (10). In conclusion, BAM-PS was chosen methodologically as it allows causal inference results which are more familiar to medical science audiences that can still be confirmed and replicated automatically through machine learning (and thus may accelerate real-time findings on larger high-dimensional datasets and data streams as they already increasingly do for other economic sectors outside of medicine), while producing more rapid and accurate results compared to traditional statistics. The more detailed rational for the use of the NIS dataset and the ML-PSr underlying BAM-PS with it (with the rationale including for its comparative advantages versus competing statistical, AI, statistical-AI hybrid, and other causal inference techniques) are documented in the above cited studies.

For this study, the propensity score for the likelihood of undergoing inpatient valvular procedure was first created (the treatment, utilizing the same above variables used in the final regression model given the double propensity score adjustment method) (13, 14), balance was confirmed among blocks, and then the propensity score was included in the final regression models as an adjusted variable. Multivariable regression was then conducted for valvular procedure, mortality, and cost, with final model performance was optimized by by backward propagation neural networks. This process was completed using a sequential prospective cohort study of patients with cardiovascular disease and cancer in a large, single center, academic medical center in the southeastern United States featuring among the world's first cardio-oncology departments (with 356 subjects from 1/2013 to 8/2020). The process was then repeated in the NIS using the above results as informative priors.

### 2.6. Model validation, reporting, and analytic software

Mean values are reported with standard deviation (SDs). Fully adjusted regression results were reported with 95% confidence intervals (CIs) with statistical significance set at a 2-tailed *p-*value of <0.05. Statistical analysis was performed with STATA 17.0 (STATACorp, College Station, Texas, USA), and machine learning analysis was performed with Java 9 (Oracle, Redwood Chores, California, USA).

## 3. Results

### 3.1. Descriptive statistics and bivariable analysis of the overall sample by MCT

Among 101,521,656 hospitalized adult patients from 2016 to 2018, 55,910 (0.06%) had MCT, of whom 13,605 (0.24%) had TVR and 8,200 (0.15%) had PVR (Table 1). Patients with MCT vs. those without had increased mortality (2.93% vs. 2.04, *p* = 0.002), longer mean length of stay (12.20 vs. 4.62 days, *p* < 0.001), and increased mean total hospitalization cost ($70,252.18 vs. 51,092.01, *p* < 0.001) (Table 2). The mean age of patients with MCT was 66.12 (SD 3.36) and the percentage of females was 56.35%. In the cohort of MCT patients, 33.60% had a history heart failure. There was a significant difference (*p* < 0.001) in payor status between MCT patients and those without MCT, with MCT more likely to have commercial insurance and less likely to have Medicare.

**Table 1:** Bivariable analysis by malignant carcinoid tumor among patients undergoing any valvular procedure (*N* = 172,587)^*^.

**Variables**	**2016–2018**			**2016**			**2017**			**2018**		
	**No-MCT**	**MCT**	***P-*** **value**	**No-MCT**	**MCT**	***P-*** **value**	**No-MCT**	**MCT**	***P-*** **value**	**No-MCT**	**MCT**	***P-*** **value**
**Variable, %**												
**Demographics**												
Age, mean (SD)	74.22 (0.16)	66.12 (3.36)	0.254	76.76 (0.15)	60.00 (4.34)	< 0.001	73.29 (0.18)	74.86 (2.07)	0.715	73.62 (0.16)	63.50 (3.66)	0.045
Female	46.92	56.35	0.290	47.05	66.67	0.239	46.63	38.10	0.433	47.07	64.29	0.197
Race			0.682			0.227			0.969			0.850
White	84.38	83.89		84.84	88.89		84.21	84.21		84.10	78.57	
Black	5.28	4.13		5.43	0.00		5.29	5.26		5.12	7.14	
Hispanic	5.72	4.13		4.81	0.00		5.83	5.26		6.53	7.14	
Asian	3.03	5.46		1.47	11.11		5.83	5.26		1.78	0.00	
Native American	0.30	0.00		0.28	0.00		0.36	0.00		0.26	0.00	
Insurance			0.001			< 0.001			0.718			< 0.001
Commercial	10.73	38.36		9.57	55.56		11.70	9.52		10.93	50.00	
Medicare	81.76	56.88		84.39	44.44		80.07	90.48		80.83	35.71	
Medicaid	4.93	4.76		3.83	0.00		5.50	0.00		5.46	14.29	
VA	1.71	0.00		1.25	0.00		1.94	0.00		1.95	0.00	
Urban ≥1 million	51.39	50.00	0.457	51.79	33.33	0.268	51.39	66.67	0.162	51.00	50.00	0.940
Region			0.353			0.443			0.256			0.359
New England	5.60	6.88		5.73	11.11		5.72	9.52		5.35	0.00	
Mid Atlantic	17.04	1.59		17.89	0.00		17.02	4.76		16.20	0.00	
East North Central	15.61	18.25		15.79	33.33		15.50	14.29		15.53	7.14	
West North Central	7.70	8.73		7.76	0.00		7.74	19.05		7.61	7.14	
South Atlantic	19.73	21.16		19.40	11.11		19.58	23.81		20.20	28.57	
East South Central	5.32	6.08		5.41	11.11		5.28	0.00		5.28	7.14	
West South Central	8.94	11.37		8.55	22.22		9.18	4.76		9.09	7.14	
Mountain	6.17	7.14		5.87	0.00		6.15	14.29		6.50	7.14	
Pacific	13.90	18.78		13.61	11.11		13.84	9.52		14.26	35.71	
**Past medical history**												
HTN	61.59	33.86	0.142	83.06	44.44	0.002	51.75	42.86	0.415	49.97	14.29	0.008
DM	17.91	20.93	0.065	18.88	19.71	0.195	17.17	21.63	< 0.001	17.68	21.46	< 0.001
CAD	1.75	0.57	< 0.001	2.23	0.79	< 0.001	1.59	0.60	< 0.001	1.43	0.33	< 0.001
Smoking	17.69	17.68	0.045	19.46	17.06	< 0.001	16.71	18.11	0.021	16.89	17.88	0.115
CHF	41.92	33.60	0.510	61.57	55.56	0.711	31.28	23.81	0.460	32.92	21.43	0.360
CKD 3-5	23.72	13.23	0.370	24.94	11.11	0.338	22.84	14.29	0.350	23.39	14.29	0.421
TV regurgitation	3.93	40.48	0.001	3.61	33.33	< 0.001	3.89	38.10	< 0.001	4.28	50.00	< 0.001
PV stenosis	0.56	10.32	0.001	0.44	0.00	< 0.001	0.71	9.52	< 0.001	0.54	21.43	< 0.001
**Replacement**												
TV	0.02	0.24	< 0.001	0.01	0.16	< 0.001	0.02	0.29	< 0.001	0.02	0.28	< 0.001
PV	0.01	0.14	0.013	0.00	0.00	0.040	0.01	0.16	< 0.001	0.01	0.25	< 0.001

* MCT, malignant carcinoid tumor; SD, standard deviation; VA, Veterans Affairs; HTN, hypertension; DM, diabetes; CAD, coronary artery disease; CHF, congestive heart failure; CKD 3-5, chronic kidney disease stage 3-5; TV, tricuspid value; PV, pulmonary valve.

**Table 2:** Bivariable analysis of valve procedures by malignant carcinoid tumor from 2016 to 2018 (*N* = 101,521,656).

**Variable, mean (SD)**	**Malignant Carcinoid Tumor-No**	**Malignant Carcinoid Tumor-Yes**	***P-*** **value**
	***N =*** **101,363,410**	***N =*** **55,910**	
**Mortality, %**	2.04	2.93	0.002
Tricuspid	5.09	12.22	0.242
Tricuspid + pulmonary	2.56	41.67	0.171
**Length of stay (days)**	4.62 (6.91)	12.20 (7.58)	< 0.001
Tricuspid	20.39 (38.49)	11.28 (8.60)	0.291
Tricuspid + pulmonary	12.23 (14.85)	9.13 (6.69)	0.485
**Total cost (United States dollars)**	51,092.01 (94,182.69)	70,252.18 (114,165.66)	< 0.001
Tricuspid	417,707.70 (480,759.27)	311,247.10 (189,524.47)	0.732
Tricuspid + pulmonary	300,317.20 (341,547.93)	350,040.73 (265,783.23)	0.561

### 3.2. Descriptive statistics and bivariable analysis of valvular pathology by year

The prevalence of MCT was mostly stable from 2016 (18,900 [0.06%]) to 2017 (19,025 [0.05%]) to 2018 (17,985 [0.05%]). Patients with MCT vs. non-MCT were more frequently diagnosed with TV regurgitation (0.58 vs. 0.24%, *p* = 0.003), PV regurgitation (0.30 vs. 0.05%, *p* = 0.001), and PV stenosis (0.07 vs. 0.01%, *p* < 0.001), but no significant significant difference for other valvular pathologies (Table 3).

**Table 3:** Descriptive and bivariable analysis of valve pathology by presence or absence of malignant carcinoid tumor by year (*N* = 101,521,656).

**Variables**	**2016–2018**				**2016**				**2017**				**2018**			
	**Sample**	**No-MCT**	**MCT**	***P-*** **value**	**Sample**	**No-MCT**	**MCT**	***P-*** **value**	**Sample**	**No-MCT**	**MCT**	***P-*** **value**	**Sample**	**No-MCT**	**MCT**	***P-*** **value**
	101,521,656	101,363,410	55,910		30,195,722	30,074,486	18,900		35,798,453	35,779,428	19,025		35,527,481	35,509,496	17,985	
**Valve pathology, %**																
**Regurgitation**																
Tricuspid	0.24	0.24	0.58	0.003	0.25	0.25	0.48	0.006	0.23	0.23	0.60	< 0.001	0.23	0.23	0.67	< 0.001
Pulmonary	0.05	0.05	0.30	0.001	0.06	0.06	0.19	0.001	0.05	0.05	0.32	< 0.001	0.05	0.05	0.39	< 0.001
Mitral	1.59	1.59	1.44	0.478	1.81	1.81	1.30	0.017	1.50	1.50	1.60	0.616	1.47	1.47	1.42	0.800
Aortic	0.34	0.34	0.35	0.379	0.38	0.38	0.42	0.628	0.33	0.33	0.21	0.201	0.32	0.32	0.42	0.309
**Stenosis**																
Tricuspid	0.00	0.00	0.02	0.263	0.00	0.00	0.03	0.045	0.00	0.00	0.00	0.743	0.00	0.00	0.03	0.002
Pulmonary	0.01	0.01	0.07	< 0.001	0.00	0.00	0.05	< 0.001	0.01	0.01	0.08	< 0.001	0.01	0.01	0.08	< 0.001
Mitral	0.03	0.03	0.05	0.608	0.03	0.03	0.05	0.486	0.03	0.03	0.03	0.954	0.03	0.03	0.06	0.385
Aortic	1.40	1.40	1.49	0.684	1.52	1.52	1.53	0.937	1.33	1.33	1.60	0.143	1.34	1.34	1.33	0.973

### 3.3. Descriptive statistics and bivariable analysis of valvular procedure by year

**Figure 1:**
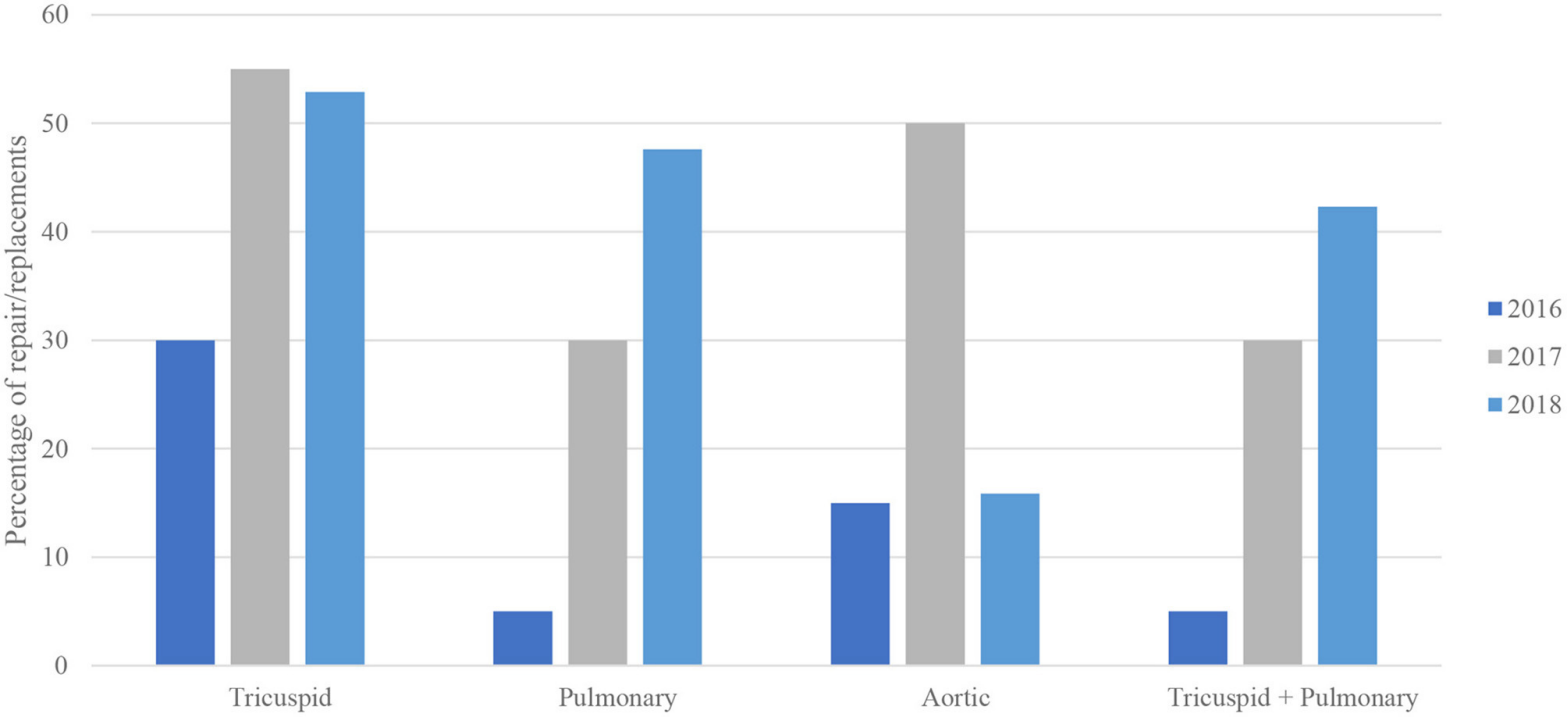
Bivariable analysis of valve procedures in malignant carcinoid tumor by year (*N* = 101,521,656).

There was a generally increased rate of valvular procedures with each subsequent year from 2016 to 2018, including with a stepwise increase in the rate of both tricuspid and pulmonary valve procedures reaching its peak in the most recent year of 2018 (Table 1 and Figure 1). For MCT vs. non-MCT, there were significanly more procedures in 2016 for TV (0.16 vs. 0.01%, *p* < 0.001) and PV (0.03 vs. 0.00%, *p* = 0.040). This trend continued in 2017 for TV (0.29 vs. 0.02%, *p* < 0.001) and PV (0.16 vs. 0.01%, *p* < 0.001), in addition to 2018 for TV (0.28 vs. 0.02%, *p* < 0.001) and PV (0.25 vs. 0.01%, *p* < 0.001).

### 3.4. Disparity analysis of valvular procedure by year

From 2016 to 2018, there were no significant disparities in the rates of valvular procedures among patients with MCT for any valve procedure (TV, PV, MV, AV) for sex, race, income, urban density, or geographic region with the exception of 2017, in which there was a regional disparity for PV, with the highest prevalence of procedures done in the Western North at 50.00% (*p* = 0.034).

### 3.5. Multivariable regression of valvular procedure, mortality, and cost

In multivariable regression, MCT vs. no MCT significantly increased the likelihood of PV regurgitation (OR 5.89, 95%CI 3.20–10.96, *p* < 0.001) in addition to TV regurgitation (OR 2.42, 95%CI 1.56–3.96, *p* = 0.001), but not mortality (OR 0.75, 95%CI 0.61–0.92, *p* = 0.089) across 2016–2018 (Table 4). But it did not not significantly increase the likelihood of inpatient TV or PV procedure. In sub-group analysis restricted to MCT, neither TV nor PV procedures significantly increased mortality, though it did, respectively, increase cost despite additional adjustment for length of stay (respectively, $141,082.30, 95% CI 27,325.52–254,839.20, *p* = 0.015; and $355,356.40, 95%CI 78,488.57–632,224.20, *p* = 0.012).

**Table 4:** Multivariable propensity score adjusted regression of valve pathology and mortality by malignant carcinoid tumor by year (*N* = 101,521,656).

**Outcomes**	**Odds ratio (OR), 95% confidence interval (CI)**, ***p*****-value**			
	**2016–2018**	**2016**	**2017**	**2018**
Pulmonary valve regurgitation	5.89, 95%CI 3.20–10.96, *p < * 0.001)	4.05, 95%CI 1.92–8.53, *p < * 0.001	5.14, 95%CI 2.67–9.93, *p < * 0.001	8.49, 95%CI 5.00–14.41, *p < * 0.001
Tricuspid valve regurgitation	2.42, 95%CI 1.56–3.96, *p =* 0.001	2.33, 95%CI 1.44–3.77, *p =* 0.001	2.14, 95%CI 1.37–3.78, *p =* 0.001	2.80, 95%CI 1.86–4.33, *p < * 0.001
Mortality	0.75, 95%CI 0.61–0.92, *p =* 0.089)	0.68, 95%CI 0.55–0.83, *p < * 0.001	0.67, 95%CI 0.54–0.83, *p < * 0.001	0.89, 95%CI 0.73–1.09, *p =* 0.265

## 4. Discussion

This is the first known multi-year nationally representative machine learning and propensity score-supported analysis of MCT and valvular pathology, procedure, disparities, mortality, and cost, in addition to being the largest known study of MCT (*n* = 55,910) according to TVR (*n* = 13,605) and PVR (*n* = 8,200). It provides novel evidence to support and clarify prior research. This includes how patients with vs. without MCT were more likely to have diagnosed TV and PV pathology along with documenting increasing rates of valvular repair and replacements (and costs but stable comparable mortatity without significant disparities, except for isolated regional disparities in 2017).

Due to the characteristic deposition of fibrous, plaque-like tissue on the endocardial surface of valvular cusps, leaflets, and subvalvular apparatus, valve replacement is the only curative treatment in patients with CHD (15–17). Additionally, valvular surgery is the only intervention demonstrating improvement in short-term survival, cardiac function, and quality of life. Medical therapy, such as diuretics, somatostatin analogs, telotristat ethyl, and peptide receptor radionuclide therapy have not been shown yet to reduce mortality or progression of CHD (17). Symptomatic patients with metastatic carcinoid tumors who do not undergo valve surgery have a limited life expectancy with a median survival of ~1 year despite treatment with somatostatin analogs (16). In our study, there is an encouraging trend toward greater TV and PV valve replacement in patients with MCT from 2016 to 2018 yet there is significant persistent concern that CHD remains underdiagnosed and its effective treatments underutilized.

Epidemiologically, the prevalence of CHD has varied from ~40 to 50% in studies from the 1980s and 1990s, to 20% in a study from 2008 (17). In our national cohort of 55,910 patients with MCT, the prevalence of TR was 58% and PR was 30%, which is more consistent with earlier studies. Medically, over the last decade there has been a parallel improvement in MCT treatment and surgical treatment specifically for CHD (18). In the largest presented surgical study of patients with CHD from Mayo Clinic, a total of 195 patients underwent TVR, with 157 (80.5%) undergoing concomitant TVR and PVR. Short-term mortality rate following valve surgery was 29% from 1985 to 1994, 7% from 1995 to 2004, 5% from 2005 to 2018, and 3.7% from 2010 to 2012 (18, 19). Following valve surgery, the patients in this series experienced notable symptomic improvement, with 85% reporting New York Heart Asssociation (NYHA) symptoms <III (and 0% reporting class IV symptoms). This has particular survival implications as only 10% of patients with greater than class II symptoms survive past 2.5 years with medical therapy alone (20).

In terms of longitudinal outcomes, patients with MCT and advanced age or NYHA functional class, right-sided heart failure and the need for pre-operative intravenous diuretic treatment have worse perioperative and long-term survival after surgical intervention for CHD (15, 21). These outcomes are in part explained by delayed diagnosis or a late decision for surgical interventions. As valve replacement for CHD has generally acceptable short-term mortality, recent focus has been on identifying appropriate candidates for earlier intervention that may improve overall survival (18).

In terms of potentially confounding clinical factors and challenges to optimizing CHD outcomes, CHD in general and PV pathology in particularl may be underrecognized and under-treated. Aalthough the TV is nearly always replaced during surgical management of CHD due to the invariable presence of TV regurgitation, PV pathology is under-diagnosis and so may contribute quietly to right heart failure and related symptoms (3–5). Echocardiographic visualization of the PV is difficult in adults with CHD, especially when there is concomitant severe TV regurgitation. If it is severe, the stroke volume reaching the PV is substantially reduced, which can reduce stenotic gradients and regurgitant volume. PV regurgitation has also been shown to directly impact right ventricular size and function after TV regurgitation.

Additionally, it can be clinically difficult to distinguish symptoms of right-sided heart failure from end-stage metastatic carcinoid disease, as both can present with progressive fatigue, edema, and ascites (22). Early identification of CHD using biomarkers (NT-proBNP level of >260 pg/ml) has sufficient sensitivity, specificity and negative predictive value for the diagnosis of CHD (22). Cardiovascular MRI (CMR) is particularly useful when echocardiography is insufficient or inconclusive in the evaluation of right-sided valvular structures (especially the PV) and right ventricular remodeling, and it is superior estimating cardiac chamber volumes and measuring volumetric forward and backward flow across valves by phase-contrast techniques (23). CMR data has demonstrated a significant reduction (~40%) of right ventricle volumes and improvement in biventricular function after PVR, while uncorrected significant PV regurgitation after TVR may lead to progressive right heart dilatation and adverse longitudinal outcomes (24). Further, it is not uncommon to see unmasking of severe PV regurgitation by the higher flow through the PV after TVR (25). Thus, PV pathology should be recognized and PVR may need to be considered before irreversible right ventricular dysfunction occurs (26). In a recent meta-analysis of 416 patients, 97% had moderate or severe TV regurgitation, of which 99% underwent TVR (while 72% had moderate or severe PV regurgitation, of whom only 59% underwent PVR) (5), suggesting needed procedural treatments may be underutilized in this patient population.

The current 2020 AHA/ACC guidelines recommend PVR as a Class I recommendation in the setting of symptomatic moderate or greater PV regurgitation and RV dilation, and a Class IIb recommendation for asymptomatic moderate or greater PR with right ventricle dilation if there is evidence of progressive right ventricular remodeling, dysfunction, and/or decline in cardiopulmonary exercise testing (27). Knowing that right heart failure can develop rapidly in patients with classic carcinoid syndrome without any relation to duration or progression of the metastasizing tumor disease, an earlier surgical intervention may be optimal. Our study provides national evidence using machine learning and propensity score-supported multivariable regression analysis that patients with and without MCT may have comparable inpatient mortality for both TVR and PVR (adjusted for known clinical confounders, though patients with MCT in general have worse mortality overall compare to those without MCT, and thus these results suggest that patients with MCT are being appropriately selected and effectively managed for TVR and PVR). Prior research has additionally suggested that valve replacement for CHD has acceptable short-term mortality, thus supporting that future research and clinical development may need to focus on better identifying appropriate candidates for earlier intervention that may improve overall survival (18).

It should be noted that there are disagreements from prior research and our present study. This analysis suggests the mortality prevalence for TVR (12.22%) and concomitant TVR and PVR (41.67%) in patients with MCT is considerably higher when compared to the Mayo Clinic study and non-MCT patients, reflecting possibly the increased impact of the small number of mortality cases, increased patient frailty from delayed indication for the procedure, and decreased expertise of low-volume centers nationally compared to a single large academic medical center. This national analysis suggests that MCT did not significantly increase the likelihood of inpatient TV or PV procedure in our study, which may reflect a conservative attitude toward TVR and PVR. Additionally, there were no significant disparities for TVR and PVR, with the exception of 2017 in which there were significantly more PVRs performed in the North Western region, suggesting referral patterns for patients with rare diseases such as CHD to high volume centers with greater technical expertise to perform these complex procedures. Together, these findings suggest that by increasing the clarity on valve replacement indications and consolidating centers of excellence of NET, the results of academic tertiary centers could potentially be extended broadly included to traditionally underserved communities. Further MCT research and dedicated societal guidelines addressing timing, perioperative management and surgical procedure standardization will be necessary to improved outcome and disparities.

In terms of study limitations, our findings should be interpreted cautiously considering the following. Our non-randomized observational study design utilizing administrative short-term inpatient data including of a smaller number of mortality cases within TVR and PVR could all limit the internal and external validity and thus generalizability of these findings. To mitigate the related biases, this study utilized a multi-year nationally representative sample from hundreds of hospitals in addition to a robust causal inference statistical methodology that included rigorous model optimization techniques including machine learning, an analytic approach repeatedly documented and utilized in the cardiolgy literature (5, 10–12).

## 5. Conclusion

Results from the largest and longest known nationally representative study of valvular pathology, procedures, mortality, and cost by MCT suggest a favorable trend clinically recognizing the need for TVR and PVR in patients with MCT, which appear safe but with increased cost compared to patients without MCT. Our study also suggests that PV pathology, traditionally suboptimally imaged and uncommonly recognized, is coming more often in focus with a favorable trend in PVR. Further research and updated societal guidelines can help sharpen needed focus on the “forgotten pulmonic valve” and improve morbidity and mortality in this often overlooked patient population.

## Data availability statement

The data analyzed in this study is subject to the following licenses/restrictions: The data is available through the United States Department of Health and Human Services by application and fee. Requests to access these datasets should be directed to HCUP Central Distributor, HCUP@AHRQ.gov.

## Ethics statement

Ethical review and approval was not required for the study on human participants in accordance with the local legislation and institutional requirements. Written informed consent for participation was not required for this study in accordance with the national legislation and the institutional requirements.

## Author contributions

Study design: DM, AB, and CI. Data collection: DM and CI. Data analysis: DM. Data interpretation, manuscript editing, and final approval: DM, AB, AJ, KM, KH, JK, RP, BA, DH, AD, EK, PK, JL-M, SY, MC, MM, IG, JY, SH, and CI. Manuscript generation: DM and AB. All authors contributed to the article and approved the submitted version.
